# Supplementary material for: Role of Bacteria-Derived Flavins in Plant Growth Promotion and Phytochemical Accumulation in Leafy Vegetables
Source: Int J Mol Sci. 2023 Aug 28;24(17):13311. doi: 10.3390/ijms241713311 (PMC10488295; doi:10.3390/ijms241713311)
Supplement: Supplementary file 1 [file ijms-24-13311-s001.zip › ijms-2559931-supplementary.pdf]

Table S1. Effects of *S. meliloti* treatment on kale (*Brassica oleracea* var. acephala) growth, physiological, and yield parameters

Treatment	Number of leaves	Plant height (cm)	Stem diameter (mm)	F_v/F_m	F_v/F_o	Fresh weight (g)
FL^+ strain	8 ± 1a	19.813 ± 1.6a	5.68 ± 0.7a	0.803 ± 0.01a	4.100 ± 0.16a	19.0 ± 1.9a
FL^- strain	8 ± 1a	19.750 ± 1.4a	5.18 ± 0.2ab	0.799 ± 0.01a	4.009 ± 0.17a	17.75 ± 1.7a
No inoculation	8 ± 1a	19.086 ± 1.5a	5.10 ± 0.4b	0.796 ± 0.01a	3.999 ± 0.29a	17.93 ± 1.9a

S. meliloti 1021: FL^+ strain; *S. meliloti* 1021Δ*ribBA*: FL^- strain; control: no inoculation. Fv/Fm: maximum quantum efficiency of PSII and Fv/Fo: potential photosynthetic capacity. Different alphabetical letters denote significant differences ($p < 0.05$) between treatment means by Fisher's least significant difference test.

Table S2. Effects of *S. meliloti* treatment on lettuce (*Lactuca sativa*) growth, physiological, and yield parameters

Treatment	Number of leaves	Plant height (cm)	Stem diameter (mm)	F_v/F_m	F_v/F_o	Fresh weight (g)
FL^+ strain	9 ± 1a	20.83 ± 1.07a	7.51 ± 0.6a	0.808 ± 0.008a	4.217 ± 0.21a	51.86 ± 2.6a
FL^- strain	9 ± 1a	19.83 ± 1.25ab	7.35 ± 0.5ab	0.806 ± 0.007a	4.172 ± 0.18a	49.33 ± 2.8a
No inoculation	8 ± 1b	18.70 ± 1.30b	6.77 ± 0.7b	0.804 ± 0.007a	4.132 ± 0.18a	41.17 ± 2.6b

S. meliloti 1021: FL^+ strain; *S. meliloti* 1021Δ*ribBA*: FL^- strain; control: no inoculation. Fv/Fm: maximum quantum efficiency of PSII and Fv/Fo: potential photosynthetic capacity. Different alphabetical letters denote significant differences ($p < 0.05$) between treatment means by Fisher's least significant difference test.

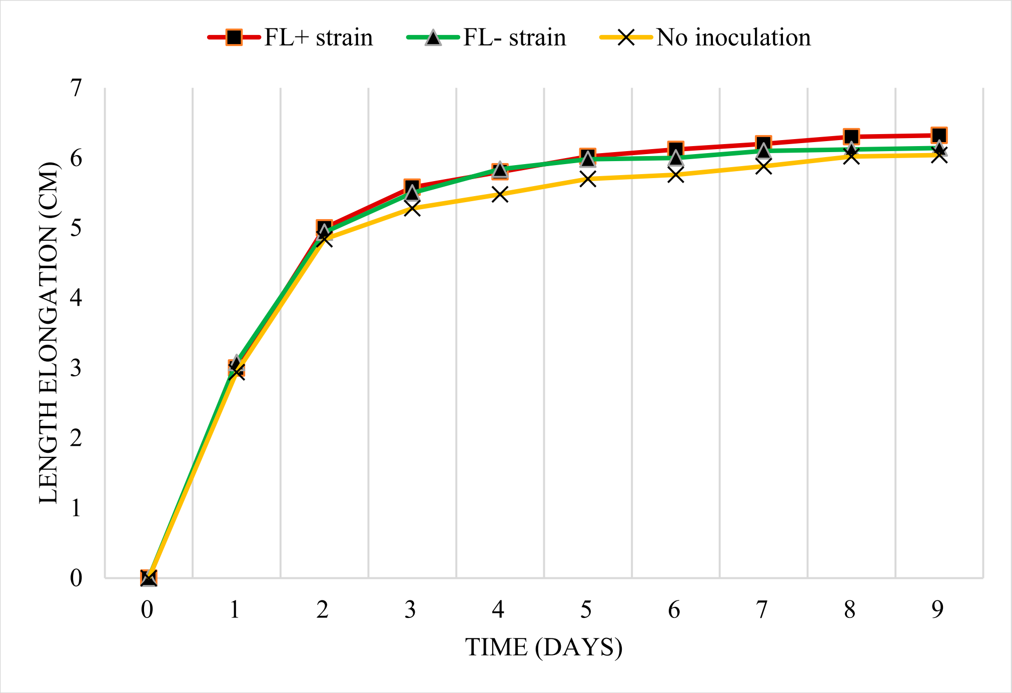

Figure S1. Effects of *S. meliloti* inoculation on kale (*Brassica oleracea* var. *acephala*) leaf elongation. *S. meliloti* 1021: FL+ strain; *S. meliloti* 1021ΔribBA: FL⁻ strain; control: no inoculation.

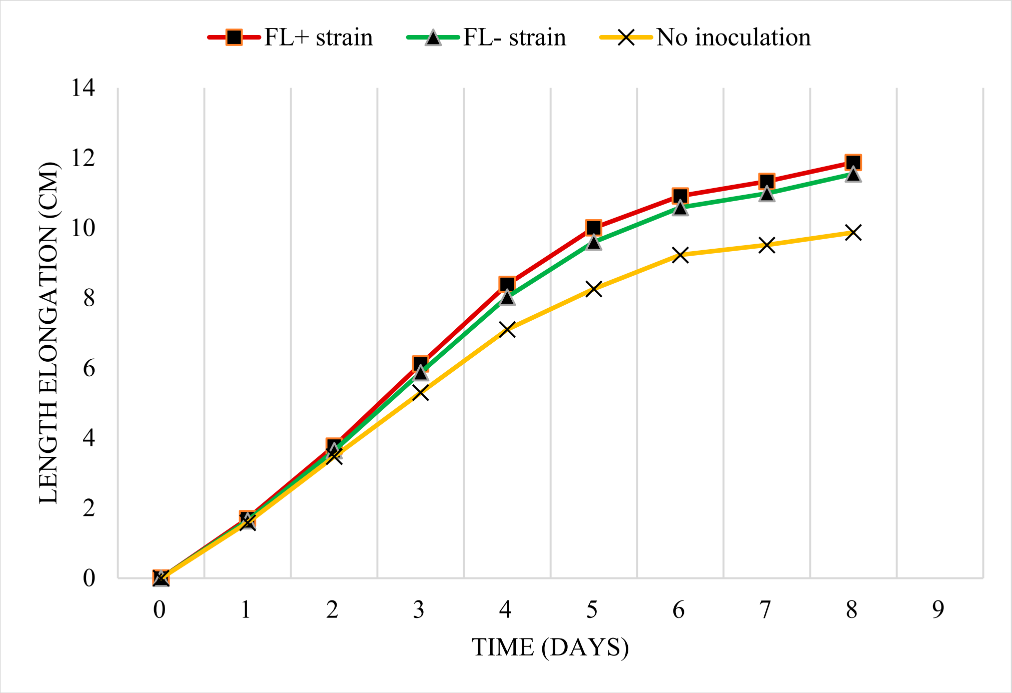

Figure S2. Effects of *S. meliloti* inoculation on lettuce (*Lactuca sativa*) leaf elongation. *S. meliloti* 1021: FL^+ strain; *S. meliloti* 1021Δ*ribBA*: FL^- strain; control: no inoculation.
